# Membranous Croup, with Report of Cases Treated with Tracheotomy

**Published:** 1893-11

**Authors:** R. M. Harbin

**Affiliations:** Calhoun, Ga.


### MEMBRANOUS CROUP, WITH REPORT OF CASES TREATED WITH TRACHEOTOMY.

BY R. M. HARBIN, M. D.

Read before the Tri-State Medical Society of Alabama, Georgia and Tennessee, at Chattanooga, October 17, 1893.

(Abstract.)

Conclusion 1.—Membranous croup is almost invariably fatal without surgical treatment, and without medicinal treatment but little can be hoped for.

2.—Any hope for an expectant plan of treatment is nil, and the few cases that recover without surgical treatment do not demand consideration.

3.—Tracheotomy is a justifiable surgical procedure and should be performed in all cases where our therapeutic measures have been exhausted and patient is in iminent danger of suffocation.

4.—It should be performed in all hopeless cases, since it either offers a chance for the patient or it promotes euthanasia.

5.—I think if the operation should happen to be performed when not absolutely necessary the patient would get well, for it is usually the complications that kill and not the operation.

6.—Statistics are misleading and do not do the operation justice, for if it was done earlier in the disease, after giving medicinal treatment a fair trial, and if we should eliminate the danger of infectious diseases as diphtheria from statistics we would have a greater per cent of recoveries. In our smaller towns we can easily exclude the diphtheritic complication where diphtheria is unknown, but in cities that is not the case.

7.—The after-treatment is more important than the operation itself.

8.—Tracheotomy keeps the patient alive until the pseudo-membrane resolves into a muco-purulent liquid and is expectorated through the tube.

9.—In all human certainty the cases I have reported would have died without the operation.

10.—That a lack of instruments is no excuse for the non-performance of the operation, for every physician can supply himself with a tube in addition to his other instruments.

As to the details of the operation I wish to emphasize the following points:

1.—That ragged edge is preferable to clean cut incisions. The tissues should be lacerated as much as possible with the point of scapel so as to prevent hemorrage.

2.—Haste makes the operation more difficult.

3.—The importance of keeping the tube constantly moistened with lime water and keeping the room at an equable temperature.

4.—That the tube should not be removed until the purulent character of the sputa ceases, which is about the 8th day.

5.—The importance of applying adhesive plasters over two small rolls of cloth, applied on either side of wound so as to press bottom sides of wound together for the first few days, thus preventing any danger of tracheal fistula.

